# Expanded DEMATEL for Determining Cause and Effect Group in Bidirectional Relations

**DOI:** 10.1155/2014/103846

**Published:** 2014-02-16

**Authors:** Elham Falatoonitoosi, Shamsuddin Ahmed, Shahryar Sorooshian

**Affiliations:** ^1^Manufacturing System Integration (MSI), Department of Mechanical Engineering, Faculty of Engineering, University of Malaya, 50603 Kuala Lumpur, Malaysia; ^2^Faculty of Technology, Universiti Malaysia Pahang, 26300 Gambang, Kuantan, Pahang, Malaysia

## Abstract

Decision-Making Trial and Evaluation Laboratory (DEMATEL) methodology has been proposed to solve complex and intertwined problem groups in many situations such as developing the capabilities, complex group decision making, security problems, marketing approaches, global managers, and control systems. DEMATEL is able to realize casual relationships by dividing important issues into cause and effect group as well as making it possible to visualize the casual relationships of subcriteria and systems in the course of casual diagram that it may demonstrate communication network or a little control relationships between individuals. Despite of its ability to visualize cause and effect inside a network, the original DEMATEL has not been able to find the cause and effect group between different networks. Therefore, the aim of this study is proposing the expanded DEMATEL to cover this deficiency by new formulations to determine cause and effect factors between separate networks that have bidirectional direct impact on each other. At the end, the feasibility of new extra formulations is validated by case study in three numerical examples of green supply chain networks for an automotive company.

## 1. Introduction

Decision-Making Trial and Evaluation Laboratory (DEMATEL) technique was proposed by Fontela and Gabus at the end of 1971 to overcome many global complex problems in scientific, political, and economic by considering experts' attitudes [1, 2]. In practice, DEMATEL method has been applied to demonstrate the interrelations among criteria and to find aspects/criteria which play central roles in system to represent the effectiveness of them [3, 4]. In addition, hybrid combination models of DEMATEL with other methods have been extensively used in different fields such as airline security evaluation [5], e-learning assessment [6], and green supplier evaluation [7]. Furthermore DEMATEL is not only used to obtain the influence levels of each element over others but also has been applied to detect complex relationships and build an impact-relation map (IRM) of the criteria [8, 9]. Combination of DEMATEL and fuzzy logic was proposed to divide required qualifications for enhanced promotion of the competency development of global managers which involves the vagueness of human judgments [10]. Then, these influence level values were adopted as the foundation of the normalization supermatrix to specify ANP weights to obtain the relative importance [11].

In general, DEMATEL technique is able to determine direct, indirect, and interdependence among the unpredictable features or attributes [12]. In addition, DEMATEL helps to identify practical solutions for particular problems and cluster of complicated problems [5, 6, 13, 14]. In DEMATEL structure, each factor or part may exert on and obtain from other higher or lower level factors [6, 7]. One of excellence of this technique rather than others decision making method in applying feedback application. The entire factors establish worth and importance of factors instead of considering only specific factors [3].

The foundation of DEMATEL is graph theory and enables us to have an enhanced realization of causal relationships by dividing important and related issues to cause and effect [15] as well as making it possible to visualize the causal relationships of subcriteria and systems in the course of causal diagram that it may demonstrate communication network or a little control relationships between individuals [8–10]. One of the most essential preferences of DEMATEL is related to visualization of the causal relationships of criteria inside networks. By dividing criteria to cause and effect group, considerable development will accrue in the whole system. Factors, which belong to the cause group, have a significant influence on system and factors that belong to the effect group. Therefore, by improving cause factors, effect factors will be developed spontaneously. On the other hand, the essential deficiency in DEMATEL technique which is noticed from its past studies was the failure to figure out cause and effect factors in bidirectional relationships between different clusters with relevant factors but detecting the cause factors is the most important part in this method because of its emphasis on developing the whole system [7].

Therefore, this study covers the bidirectional relationships and interactions among the potentially significant factors and their direct and indirect effects on each other and visualizes the causal relationships among criteria and subcriteria in a system by creating a structural relationship map in three different green supply chain networks. For this purpose, a new formulation known as an “expanded DEMATEL” technique is applied. The feasibility of the new formulations has been examined in the biggest automotive company in Middle East which is called Iran Khodro. The research enables researchers to determine the cause and effect factors in bidirectional relations in networks by the expanded DEMATEL when the number of rows is not equal to the number of columns between different clusters which consist of several factors. The newly expanded approach is illustrated with three case study examples.

The rest of the study is structured as follows.  Section 2 presents the primary edition of DEMATEL.  Section 4 identifies the infeasibility of the original DEMATEL to find cause and effect factors in bidirectional relationships between different clusters/criteria. In Section 5, expanded DEMATEL is proposed. Three numerical/empirical examples to validate the expanded DEMATEL are applied in Section 3. Finally conclusion is presented in Section 6.

## 2. The Primary DEMATEL

For clarification, the primary DEMATEL technique is explained as follows.

**Step 1 (find the direct relation (average) matrix A)**

Suppose that there are *n* factors to be considered and *H* experts to provide their opinions. Each expert is asked to specify the degree to which he or she believes that the factor *i* influences the factor *j*. This pairwise comparison between the *i*th factor and the *j*th factor given by *k*th expert is indicated as *x*_*ij*_^*k*^ which takes an integer score ranging between 0, 1, 2, 3, and 4, representing “no influence (0),” “low influence (1),” “medium influence (2),” “high influence (3),” and “very high influence (4),” respectively. The scores are given by each expert and *X* ^1^ *X* ^2^ ⋯ *X* ^*H*^ are answers of each of them that make the *n* × *n* nonnegative matrix *X* ^*k*^ = [*x*_*ij*_^*k*^]_*n*×*n*_ with 1 < *k* < *H*. A high score indicates a belief that greater improvement in *i* is required to improve *j*. The diagonal elements of each answer matrix *x*_*ij*_^*k*^ are all set to zero, which means no influence is given by itself. Then it is possible to calculate the *n* × *n* average matrix **A** on account of all experts opinions by averaging the *H* of their scores as follows:

(1) $$\begin{array}{c} {\left[a_{ij}\right]}_{n\times n}=\frac{1}{H}\overset{H}{\underset{k=1}{\sum }}{\left[x_{ij}^{k}\right]}_{n\times n}. \end{array}$$

**Figure 1:**
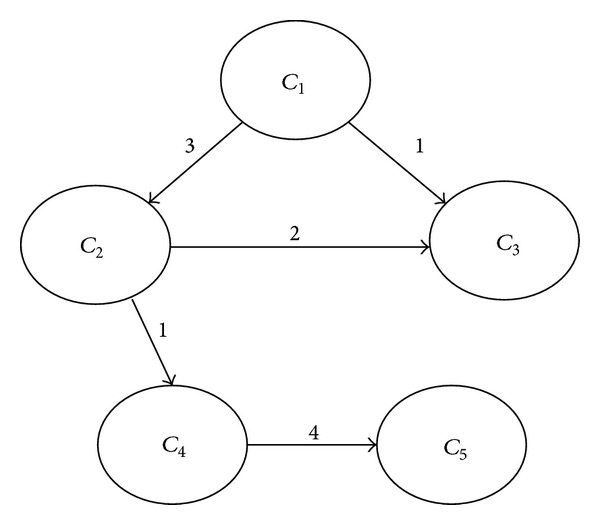
Example of an influence map.

The average matrix **A** = [*x*_*ij*_^*k*^]_*n*×*n*_ shows the initial direct effects that a factor exerts on and receives from other factors; hence, the initial direct relation matrix has been called. In addition, the causal effect between each pair of factors in a system can be mapped out by drawing an influence map.  Figure 1, indicates an example of an influence map. Here, each node corresponds to a factor in the system and arrows represent impacts between factors. As an instance, an arrow from *C*_1_ to *C*_2_ shows the influence that *C*_1_ exercises on *C*_2_, and the strength of its effect is three. DEMATEL is able to convert the structural relations between the factors of a system into an intelligible map of the system.

**Step 2 (determine the normalized initial direct relation matrix D)**

By normalizing the average matrix **A**, normalized initial direct relation matrix **D** = [*d*_*ij*_]_*n*×*n*_ is obtained in the following formula:

(2) $$\begin{array}{c} S=\max\left\{\underset{1\leq i\leq n}{\max}\overset{n}{\underset{j=1}{\sum }}aij,\underset{1\leq j\leq n}{\max}\overset{n}{\underset{i=1}{\sum }}aij\right\},\\ D=\frac{A}{S}. \end{array}$$

Consequently, ∑_*j*=1_^*n*^ *a* *ij* represents total direct effects that criterion *i* gives to the other criteria that are obtained by sum of each row *i* of matrix **A** as well as the sum of each column *j*; ∑_*i*=1_^*n*^ *a* *ij* represents total direct effects received by creation *j*. In addition, max⁡_1≤*i*≤*n*_∑_*j*=1_^*n*^ *a* *ij* represents the largest total direct effect of all factors and max⁡_1≤*j*≤*n*_∑_*i*=1_^*n*^ *a* *ij* represents the largest total direct effect received for all factors. The positive scalar *s* takes the larger of the two as the scaling factor, and the matrix **D** is obtained by dividing each element of **A** by the scalar *s*. Note that each element *d*_*ij*_ of matrix **D** is between zero and one [16].

**Step 3 (calculate the total relation matrix T)**

*D* ^*m*^ is the power of the normalized initial direct relation matrix **D** which is identified as a *m*-indirect effect and can be used to demonstrate the effect of length *m* or the effect propagated after *m* − 1 intermediates [16]. A continuous reduction of the indirect effects of problems besides the powers of matrix **D**, like an engrossing Markov chain matrix, guarantees convergent solutions to the matrix inversion. The total influence or total relation can be obtained by summing up

(3) $$\begin{array}{c} D^{2},D^{3},\ldots ,D^{\infty },\\ \underset{m\to \infty }{\lim}D^{m}={\left[0\right]}_{n\times n},\\ {\left[0\right]}_{n\times n}\;\text{is}\;\text{a}\;n\times n\;\text{null}\;\text{matrix}. \end{array}$$

The total relation matrix **T**_*n*×*n*_ is achieved as follow:

(4) ∑m=1∞Di=D+D2+D3⋯Dm=D(I+D+D2+⋯+Dm−1)=D(I−D)−1(I−D)(I+D+D2+⋯+Dm−1)=D(1−D)−1(I−Dm)=D(I−D)−1.

*I* is identity matrix. *T* is total relation matrix ([**T**]_*n*×*n*_).

The sum of rows and sum of columns of the total relation matrix **T** are computed as **r** and **c** *n* × 1 vectors

(5) $$\begin{array}{c} {\left[r_{i}\right]}_{n\times 1}={\left(\overset{n}{\underset{j=1}{\sum }}t_{ij}\right)}_{n\times 1}, \end{array}$$

(6) $$\begin{array}{c} {\left[c_{j}\right]}_{1\times n}={\left(\overset{n}{\underset{i=1}{\sum }}t_{ij}\right)}_{1\times n}. \end{array}$$

The sum of the *i*th row of the matrix **T** is [*r*_*i*_]_*n*×1_ and indicates the total effects, both direct and indirect, given by the factor *I* to other factors. Likewise, [*c*_*j*_]_1×*n*_ is the sum of the *j*th column of the matrix **T** and donates the total effects, both direct and indirect, received by the factor *j* from other factors. Consequently, when *j* = *i*, the sum (*r*_*i*_ + *c*_*j*_) which is called “prominence” gives us an index representing the total effects both given and received by the factor *i*. In other words, (*r*_*i*_ + *c*_*j*_) demonstrates the degree of the importance (total sum of the effects given and received) that the factor *i* plays in the system. In addition, the difference (*r*_*i*_ − *c*_*j*_) which is called “relation” shows the net effect that the factor *i* contributes to in the system. When (*r*_*i*_ − *c*_*j*_) is positive, the factor *i* is a net causer and when (*r*_*i*_ − *c*_*j*_) is negative the factor *i* is a net receiver [6, 16].

## 3. Methodology and Case Study to Validate a New Extra Formulation

To demonstrate the validity of the expanded DEMATEL three different intelligible networks in green supply chain area are proposed. In addition, these networks as a case study with experts' interview technique are applied for this research. Ten (10) professional experts who are involved in supply chain activities in Iran Khodro Co. are interviewed. Interview has been done individually with each expert and it took between 45 and 60 minutes. During the interviews, pairwise comparisons between criteria are determined. It means that the committee with experts determines the relations between the influential factors in each of the causal evaluation networks. Each expert performs the score from 0 to 4 according to their experiences and believes that, for example, factor *i* affects factor *j*. For this purpose, a group of engineers is selected from the company's supply chain department, Sapco (Supplying Automotive Parts CO), which is the most important supplier and the main subset for Iran Khodro Company. In fact, to apply DEMATEL technique, using expert's opinion among and within the elements to a paired comparison analysis is required [17–19].

## 4. Infeasibility of Primary DEMATEL to Determine Cause and Effect in Two-Way Relations

**Example 1**

**Figure 2:**
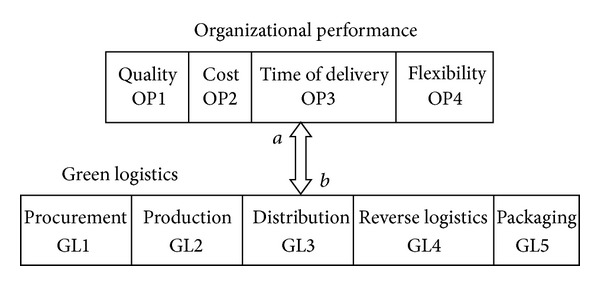
Conceptual network 1: “Organizational Performance” and “Green Logistics”.

Suppose that one conceptual network 1 which consists of two main criteria such as “Organizational Performance” (OP) and “Green Logistics” (GL) and related subcriteria is proposed for evaluation of green supplier selection as shown in Figure 2. According to Figure 2, there is a bidirectional relationship between two main criteria. “*a*” means that factors of OP have direct relations on factors of GL and “*b*” means that factors of GL have direct relations on factors of OP. Based on (1) to (4), initial direct matrix **A**, direct matrix **D**, and total relation matrix **T** are calculated for both “*a*” and “*b*” to find cause and effect factors among this relation. Calculations for relation “*a*” are as follows:

(7) Aa=[GL1GL2GL3GL4GL5OP13.63.51.432.5OP23.13.62.30.52.6OP32.73.42.60.50.90.8OP43.133.30.91.6],Da=[GL1GL2GL3GL4GL5OP10.2570.2500.10.2140.179OP20.2210.2570.1640.0360.186OP30.1930.2430.1860.0640.057OP40.2210.2140.2360.0640.114],Ta=(GL1GL2GL3GL4GL5ropOP10.9561.0040.5950.5270.6313.713OP20.7910.8790.560.2920.5563.078OP30.7660.8690.5890.3170.4252.966OP40.8360.8860.6690.3390.5053.235CGL3.3493.6382.4131.4752.117).

To determine cause and effect factor, (*r*_*i*_ + *c*_*j*_) and (*r*_*i*_ − *c*_*j*_) need to be calculated when *i* = *j*, but here it is obvious that *i* ≠ *j*. Infeasibility of primary DEMATEL has been revealed by this example. By the same way, for the relation “*b*” we have

(8) Ab=[OP1OP2OP3OP4GL13.53.41.51.7GL23.63.73.13.7GL31.42.63.32.7GL41.10.40.70.3GL532.80.30.3],Db=[OP1OP2OP3OP4GL10.2480.2410.1060.121GL20.2550.2620.2200.262GL30.0990.1840.2340.191GL40.0780.0280.050.021GL50.2130.1990.0210.021],Tb=(OP1OP2OP3OP4rLGLG10.6690.6770.4560.4762.278LG20.7730.8010.670.7082.952LG30.4460.5540.5590.5072.066LG40.1780.1340.1350.160.607LG50.5220.5170.2660.2761.581COP2.5882.6832.0862.127).

As mentioned before, here also *i* ≠ *j* and calculating (*r*_*i*_ + *c*_*j*_) and (*r*_*i*_ − *c*_*j*_) is not possible and logical. To cover this deficiency, an expanded DEMATEL will be proposed in next section.

## 5. Expanded DEMATEL

**Figure 3:**
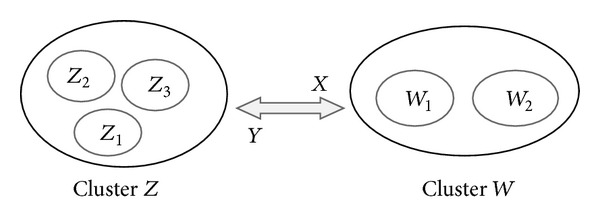
Example of bidirectional relation when *i* ≠ *j*.

According to DEMATEL technique, when =*j*, [*R*_*i*_]_*n*×1_ = (∑_*j*=1_^*n*^ *t*_*ij*_)_*n*×1_ (total given effects by factor *i*) and [*C*_*j*_]_1×*n*_ = (∑_*i*=1_^*n*^ *t*_*ij*_)_*n*×1_ (total received impacts by factor *j*) and therefore (*R* + *C*) and (*R* − *C*) will be achieved. But when *i* ≠ *j* and components in rows are different from components in columns, calculating (*r*_*i*_ + *c*_*i*_) and (*r*_*i*_ − *c*_*i*_) is not possible. As an instance, in Figure 3, relation *X* means cluster *W* and its dimensions have direct effect on cluster *Z*. Therefore, in total relationship matrix *X*, *R*_*W*_ demonstrates the total effects, both direct and indirect, given by two factors of *W* cluster to the three factors of *Z* cluster; similarity *C*_*Z*_ represents total effects, direct and indirect, received by factors of *Z* cluster from the three factors of *W* cluster:

(9) $$\begin{array}{c} {\left[{\left[R_{2}\right]}_{2\times 1}={\left(\overset{3}{\underset{j=1}{\sum }}t_{23}\right)}_{2\times 1}\right]}_{X},\\ {\left[{\left[C_{3}\right]}_{1\times 3}={\left(\overset{2}{\underset{i=1}{\sum }}t_{23j}\right)}_{1\times 3}\right]}_{Y}. \end{array}$$

In addition, relation *Y* means cluster *Z* and its dimensions have direct effect on cluster *W*. Therefore, in total relationship matrix *Y*:

(10) $$\begin{array}{c} {\left[{\left[R_{3}\right]}_{3\times 1}={\left(\overset{2}{\underset{j=1}{\sum }}t_{23}\right)}_{3\times 1}\right]}_{Y},\\ {\left[{\left[C_{2}\right]}_{1\times 2}={\left(\overset{3}{\underset{i=1}{\sum }}t_{23j}\right)}_{1\times 2}\right]}_{X}. \end{array}$$

Consequently, (*R*_*w*_ + *C*_*z*_) and (*R*_*w*_ − *C*_*z*_) (type in equation mode) are not possible (because *i* ≠ *j*), logical, and acceptable in mathematical analysis. Therefore, in past studies, researchers have not achieved cause and effect group in these kinds of relations. But in a comprehensive DEMATEL, when two matrixes in a bidirectional relation which are the same as *XY* in Figure 3 will be mixed, prominence and relation can be achieved:

(11) $$\begin{array}{c} {\left(R+C\right)}_{W}=R_{W}+C_{W},\\ {\left(R-C\right)}_{W}=R_{W}-C_{W}. \end{array}$$

As a result, by attention to result of (*R* − *C*), dimensions will be divided into cause and group; also the amount of (*R* + *C*) presents the most important factors in bidirectional relations.

Consequently, all factors of the network can be divided into cause and effect group by applying the new formulations of expanded DEMATEL as follows.

 Relation *a*:
  (12) (R+C)OP=ROP+COP,(R−C)OP=ROP−COP.
 Relation *b*:
  (13) (R+C)GL=RGL+CGL,(R−C)GL=RGL−CGL.

In bidirectional relations, logically one of criteria and its related factors are more powerful than the other one.

It means that the stronger criteria should be located as a cause group.  Table 1 indicates results of expanded DEMATEL for relations *a* and *b*.

(14) Note  that {if  (ri−ci)≥0,factor  belongs  to  cause  group,if  (ri−ci)≤0,factor  belongs  to  effect  group.

According to Table 1, all amount of (*R* − *C*)_OP_ ≥ 0; therefore organizational performance is more powerful than green logistics inside network 1 and it plays the fundamental roles in the whole system based on this bidirectional relation. To improve the system, organizations need to focus on OP and its related factors; hence, factors of GL will be developed automatically because they are affected by factors of OP. Furthermore, the highest value of (*r*_*i*_ + *c*_*i*_) belongs to GL2 and it means that GL2 is the most important factor inside the network and need more attention by organization.

**Example 2**

**Figure 4:**
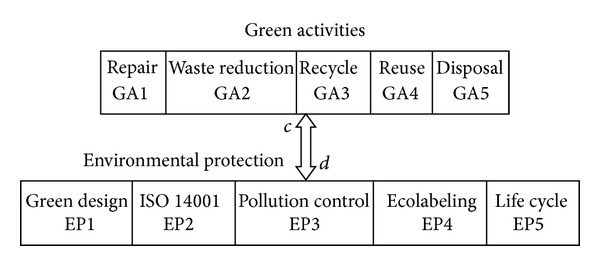
Conceptual network 2: green activities and environmental protection.

The case of *i* = *j*, when factors belong to two different brands: Figure 4 demonstrates that network 2 includes two main clusters as green activities (GA) and environmental protection (EP) with equal number of related factors: *i* = 5 and *j* = 5.

Same as Example 1, based on (1) to (4), initial direct matrix **A**, direct matrix **D**, and total relation matrix **T** are calculated for both “*c*” and “*d*” to find cause and effect factors among this relation. Calculations for relation “*c*” are displayed as follows:

(15) Ac=[EP1EP2EP3EP4EP5GA13.70.52.12.23.3GA23.61.53.92.63.2GA30.30.23.40.80.3GA42.202.310GA500.20.80.90],Dc=[EP1EP2EP3EP4EP5GA10.2500.0340.1420.1490.223GA20.2430.1010.2640.1760.216GA30.020.0140.2300.0540.020GA40.14900.1550.0680GA500.0140.0540.0610],Tc=(EP1EP2EP3EP4EP5RGAGA10.420.0650.3650.2820.3381.47GA20.4540.1440.5710.3450.361.874GA30.0620.0240.3410.0950.0460.568GA40.2370.0140.2810.1340.0620.728GA50.0240.0180.0980.0790.0110.23CEP⁡1.1970.2651.6560.9350.817).

Same as before, to determine cause and effect factor, (*r*_*i*_ + *c*_*i*_) and (*r*_*i*_ − *c*_*i*_) need to be calculated when *i* = *j*, but here the type of *r*_*i*_ and *c*_*i*_ is different. Infeasibility of primary DEMATEL will be covered with new formulations of expanded DEMATEL. By the same way, for the relation “*d*” we have

(16) Ad=[GA1GA2GA3GA4GA5EP13.63.42.41.83.2EP22.63.72.21.92.7EP31.33.23.52.30.9EP43.63.63.21.63EP53.43.92.61.40],Dd=[GA1GA2GA3GA4GA5EP10.2020.1910.1350.1010.180EP20.1460.2080.1240.1070.152EP30.0730.1800.1970.1290.051EP40.2020.2020.1800.0900.169EP50.1910.2190.1460.0790],Td=(AG1AG2AG3AG4AG5REP⁡EP10.6790.7870.590.4080.5212.985EP20.5780.7510.5390.3870.4632.718EP30.4290.6390.5570.3710.3172.313EP40.6910.8180.6530.410.523.092EP50.5640.6920.510.3280.2882.382CAG2.9413.6872.8491.9042.109).

Let us apply expanded DEMATEL to find cause and effect factor of network 2.

 Relation *c*:
  (17) (R+C)AG=RAG+CAG,(R−C)AG=RAG−CAG.
 Relation *d*:
  (18) (R+C)EP⁡=REP⁡+CEP⁡,(R−C)EP⁡=REP⁡−CEP⁡.

Table 2 illustrates results of expanded DEMATEL for relations *c* and *d* in the network 2.

(19) Note  that {if  (ri−ci)≥0,factor  belongs  to  cause  group,if  (ri−ci)≤0,factor  belongs  to  effect  group.

According to the result of Table 2, environmental protection belongs to the cause group because the amounts of (*r*_*i*_ − *c*_*i*_) ≥ 0 for all its factors. Green activities are the effect group because the amount of (*r*_*i*_ − *c*_*i*_) ≤ 0 for all its dimensions. Furthermore, the highest value of (*r*_*i*_ + *c*_*i*_) is related to the EP2. It means that EP2 plays the central role inside the network.

In both examples, validity of expanded DEMATEL has been tested and expected and logical results have been achieved. All factors of effect group have been less than zero and all factors, which belong to cause group, have been positive.

## 6. Conclusion

The current research improves DEMATEL by proposing an approach for addressing the infeasibility issue in the primary DEMATEL method which has been widely applied in many complex networks and applications. The study prove that the infeasibility might occur in networks which include bidirectional relations and then improve the method so that infeasibility can be avoided. New formulations have been added to primary DEMATEL and two case studies were provided as a proof to evidence that the expanded DEMATEL is sound and applicable to all bidirectional relations of all networks. Expanded DEMATEL can provide a feasible solution for both cases that are infeasible or feasible for the primary method because it yields a solution which is very close to that of the original DEMATEL. Expanded DEMATEL is very applicable and useful for all kinds of networks that are inclusive bidirectional relations because of determining cause and effect factor to improve the system. The new formulations provide a more comprehensive approach for those applications to which primary DEMATEL is applied.

## Figures and Tables

**Table 1:** Result of expanded DEMATEL for network 1.

*R*_a_			*R*_b_		
(*R* + *C*)_OP_		(*R* − *C*)_OP_	(*R* + *C*)_GL_		(*R* − *C*)_GL_
Cause group			Effect group		
OP1	6.301	1.125	GL1	5.627	−1.071
OP2	5.761	0.395	GL2	6.59	−0.686
OP3	5.052	0.88	GL3	4.479	−0.347
OP4	5.362	1.108	GL4	2.082	−0.868
—		—	GL5	3.698	−0.536

**Table 2:** Result of expanded DEMATEL for network 2.

*R*_c_			*R*_d_		
(*R* + *C*)_AG_		(*R* − *C*)_AG_	(*R* + *C*)_*EP*⁡_		(*R* − *C*)_*EP*⁡_
Effect group			Cause group		
AG1	2.667	−1.471	EP1	5.926	1.788
AG2	2.139	−1.813	EP2	6.405	2.453
AG3	2.224	−2.281	EP3	5.162	0.657
AG4	1.663	−0.241	EP4	4.996	2.157
AG5	1.047	−1.879	EP5	4.491	1.565
